# Enacting Plant-Inspired Robotics

**DOI:** 10.3389/fnbot.2021.772012

**Published:** 2022-01-31

**Authors:** Jonny Lee, Paco Calvo

**Affiliations:** Minimal Intelligence Laboratory (MINTLab), University of Murcia, Murcia, Spain

**Keywords:** soft robotics, embodied robotics, plant intelligence and behavior, enactivism, autonomy, growbots

## Abstract

Plants offer a source of bioinspiration for soft robotics. Nevertheless, a gap remains in designing robots based on the fundamental principles of plant intelligence, rooted in a non-centralized, modular architecture and a highly plastic phenotype. We contend that a holistic approach to plant bioinspiration—one that draws more fully on the features of plant intelligence and behavior—evidences the value of an enactivist perspective. This is because enactivism emphasizes not only features of embodiment such as material composition and morphology, but also autonomy as an important aspect of plant intelligence and behavior. The enactivist sense of autonomy concerns the dynamics of self-producing systems (such as plants) that create a distinction between themselves and a domain of interactions that bear on the conditions of viability of the system. This contrasts with the widespread, but diluted notion of autonomy that merely indicates the independent operability of a system for an arbitrary period. Different notions of autonomy are relevant for soft roboticists, for instance, when evaluating limitations on existing growing robots (“growbots”) that take bioinspiration from plants, but depend on a fixed source of energy and material provided by an external agent. More generally, plant-inspired robots serve as a case study for an enactivist approach to intelligence, while, correspondingly, enactivism calls attention to the possibility of non-zoological forms of intelligence embodied in a self-organizing, autonomous system.

## Introduction

Plants offer a rich source of bioinspiration for soft robotics. Despite progress in selected areas (see Mazzolai et al., 2020, for a mini-review), a gap remains in designing systems based on the fundamental principles of plant intelligence. More “holsitically” plant-inspired robots would inhabit bodies that exhibit a fuller range of plant features, rooted in a decentralized and modular architecture coupled with a highly plastic phenotype (Calvo et al., 2020; Calvo and Trewavas, 2021). In addition to plant-like bodies, realizing key characteristics of plant intelligence, such as flexible and adaptive growth, may require attention to the role of biological autonomy. Given its consideration of embodied features such as material composition and morphology as well as adaptive autonomy, this article indicates that the project of designing more fully plant-like systems forms a fruitful two-way exchange with enactivism (Varela et al., 1991/2017; Noë, 2004; Stewart et al., 2010; Thompson, 2010; Hutto and Myin, 2012; Di Paolo et al., 2017).

The prospect of more holistically plant-inspired robots connects with a general embodied perspective that recognizes the value of intelligent problem-solving via adaptive morphology [as demonstrated, for example, exemplar “passive dynamic walker” by McGeer (1990); for discussion, see Clark, 1997]. Smart embodiment is evidently key to plant intelligence and behavior; for instance, the material and structural properties of plant bodies are adapted to exploit physical constraints (friction, gravity, and inclination) for growth (as opposed to locomotion) (Lopez et al., 2014; Vandenbrink and Kiss, 2019). Correspondingly, plant-inspired robots indicate alternative means of adaptive embodiment in the form of growing robots or “growbots” (Laschi et al., 2016; Sadeghi et al., 2017; Del Dottore et al., 2019), i.e., systems that *move* by lengthening or extending the surface area of their bodies.

Beyond these basic considerations of embodiment, an enactive perspective also draws attention to a strong sense of autonomy, grounded in the concept of autopoiesis (Vernon, 2010). As such, enactivism can play a heuristic role in drawing attention to strong biological autonomy and reminding us that materials and morphology do not exhaust the possibilities of bioinspiration. As it pertains to plant-inspired robotics, this perspective can be used (among other things) to evaluate limitations on existing growbots, which take bioinspiration from plants, but depend on a fixed source of energy and material provided by an external agent. More broadly, considering autonomy as part of a soft and embodied perspective may serve in the development of holistically plant-like robots, while testing principles of non-animal intelligence and behavior gleaned from applying tools from plant cognitive science/neurobiology (Baluška et al., 2006a,b).

## Existing Plant-Inspired Robots and the Nature of Plant Intelligence

Existing bioinspired robots demonstrate the practical value of considering plant capacities for intelligent behavior. Recent advances in material composition, kinematic principles, and morphological features build on plant research. For example, effective adhesive mechanisms have been drawn from examinations of climbing plants, soft spiral grippers from twinning plants (Yang et al., 2020), and grasping-by-coiling behaviors from plant circumnutation—a term coined by Darwin (1875) that refers to the helical movements created by growing tips and other plant organs. Moreover, robotic growth via root-like filament deposition has taken inspiration from the plant kingdom (Blumenschein et al., 2020; Fiorello et al., 2020; Mazzolai et al., 2020).

Much of this existing plant-inspired research falls within the field of soft robotics, which is vital for understanding the holistic plant-inspired robotics targeted in this article. By “holistic plant-inspired robotics,” we refer to the development of systems that are more fully plant like in their intelligence and behavior (in a sense to be specified shortly), as opposed to merely borrowing a small number of specific materials or gadgets. Soft robotics refers to the design and construction of systems with flexible bodies using compliant materials, often drawing on the properties of living organisms (Kim et al., 2013; Calisti et al., 2017; Thieffry et al., 2017; Rich et al., 2018; Drotman et al., 2021). A common advantage of soft (over hard) robots is greater bodily flexibility and adaptability to the environmental constraints. Soft robotics, in turn, overlaps with “embodied” perspectives, introduced earlier. While soft robotics focuses specifically on the problem-solving potential afforded by compliant materials of the sorts exploited by nature (Trivedi et al., 2008), embodied perspectives more broadly draw insight from the capacities of the adaptive morphology of an organism (Hoffmann and Pfeifer, 2018). In keeping with a soft and embodied perspective, research in plant intelligence indicates the distributed nature of control and processing, where adaptive responsibility is shared between internal signaling channels, the material properties of (soft) organs, and the dynamics of body-environment interactions.

By examining existing plant-inspired robots, we can distinguish between systems that selectively borrow elements of plant design vs. systems based on the fundamental organizing principles of plant intelligence (Frazier et al., 2020). There is a spectrum. However, plant-inspired robotics has hitherto concentrated on a small number of tools for solving certain problems (although see Blumenschein et al., 2020, for instance, on designing more plant-like systems of control). As such, there remain unexplored avenues for engineering systems that manifest the full suite of fundamental features of plant intelligence. Such systems not only contain a few plant-like gadgets, but resemble plants in their basic organization.

Of course, plants exhibit as much variety in their anatomical and physiological details as animals. We should, therefore, remain sensitive to potential diversity in plant intelligence and behavior. Nevertheless, we can identify some generic principles that typify the plant kingdom, much as we can with animals (aardvarks, albatrosses, and alligators share similar centralized neural hardware and locomotion-based sensorimotor competencies, despite their myriad differences). Indeed, attending to the common character underlying plant particularities might help us to appreciate the gaps left by plant-inspired robotics that focuses only on specific bodily gadgets. The key features of plant behavior and intelligence that we take to be instructive for soft roboticists include the following:

**Distributed coordination:** Higher plants are characterized by a highly globalized yet decentralized, i.e., distributed architecture, with replicating modules that consist of branch roots (below ground) alongside leaves and subtended buds (above ground), flexibly distributed to optimize the procurement of energy and mineral resources (Calvo and Trewavas, 2021). The important point, for our purposes, is that plants display highly localized activity, while using feedback and feedforward mechanisms (Calvo and Friston, 2017) to provide stability and flexible responses to achieve organism-level adaptive behavior.

**Movement via growth:** Plants move by growth rather than locomotion (Darwin and Darwin, 1880). In animals, growth principally concerns the development of the organism as it matures and is relatively determined. In plants, growth is associated with the continuous, dynamic interaction of the organism with the environment, throughout its life, and is highly plastic. It is primarily characterized by the extension from the tip of the body (apical extension) and length change, allowing organisms to move through spatially constrained environments and adopt three-dimensional structures. Growth, thus, closely overlaps with “remodeling” of a plant, changing its material properties, and “morphogenesis,” changing its shape, to adaptively act within its dynamic environment (Del Dottore et al., 2018). Notably, as an efficient strategy for movement, growth is found across scales of natures and different kingdoms—for example, in fungal hyphae as well as networks of neurons—and is associated with the flexible exploration of three-dimensional (3D) space in a non-deterministic body (Blumenschein et al., 2020).

**Neural-like properties:** Plants lack neurons. Nevertheless, growing research highlights related molecular-level functional similarities between animal and plant substrates (Baluška and Levin, 2016; Miguel-Tomé and Llinás, 2021). One example is the fact that plants possess neurotransmitters [acetylcholine, glutamate, dopamine, histamine, noradrenaline, serotonin, and gamma-aminobutyric acid (GABA)], some of which appear to play roles analogous to those in animals (Baluška and Mancuso, 2009a; Baluška, 2010). Another example is the capacity for plant cells to produce electric potentials and exploit auxin-secreting neuron-like plant synapses (Baluška and Mancuso, 2009b). Electrical signals are transmitted along vascular conduits via networks of phloem, xylem, and cambium, again highlighting the importance of the vascular system for whole-body integration (Baluška et al., 2006).

**Swarm intelligence:** Swarm intelligence refers to the activity of the decentralized group of individuals that collectively results in the emergence of adaptive behavior. Examples include bird flocking, microbial organization, ant colony coordination, and fish schooling. Research suggests that swarm intelligence might apply to the plant roots too: local interactions between relatively simple components (root tips) result in the emergent functionality. For instance, Ciszak et al. (2012) argue that coordinated activity among individual root apices, which change in growth direction produces their episodic patterns of coordinated activity, resulting (collectively) in resource optimization.

Through their modular architecture within a highly plastic phenotype, plants engage in a range of flexible and information-sensitive capacities. Commonly observed capacities include perception, communication, kin recognition, decision-making, anticipation, learning, risk sensitivity, and mimicry (Calvo, 2016; Segundo-Ortin and Calvo, 2021). Plants, thus, display remarkably intelligent behaviors without the need for a central control organ.

### Enacting Bioinspiration

As our discussion so far suggests, designing systems that are more fully plant-like accords with soft robotics and a broader embodied perspective. One reason for this emphasis on soft bodies and smart morphology is that plant intelligence lacks the sort of organization and architecture modeled by symbolic, language like, or more explicitly deliberative architectures (Newell and Simon, 1976; Pylyshyn, 1984). Research in plant intelligence, for instance, indicates the distributed nature of control, where adaptive responsibility is shared between local responses, internal long-distance signaling mechanisms, the material properties of organs, and the dynamics of body-environment interactions (recalling the “principle of ecological balance,” Pfeifer and Scheier, 1999). More fully plant-like robots will exploit similar means for adaptive behavior through principles of the smart embodiment such as sensorimotor coupling with soft bodies, and decentralized control (Linson and Calvo, 2020; Calvo and Trewavas, 2021).

Enactivism stresses the role of an adaptive embodiment for intelligence and behavior and, thus, coheres with other soft and embodied perspectives, but additionally centers the role of “autonomy” and “adaptivity” (Froese and Ziemke, 2009), based on the conviction of strong continuity between life and mind (Varela et al., 1991/2017; Thompson, 2007). As with all organisms, such adaptive autonomy plausibly plays an important role in plant intelligence and behavior, as we shall see. We contend, therefore, that an enactive perspective on plant bioinspiration serves as a heuristic for drawing attention to the contribution of soft materials and morphology to plant intelligence as well as ask us to consider the role of adaptive autonomy. On the flipside, plant bioinspiration offers enactivism a case study for exploring the possibility of engineering more fully agential systems.

Enactivism refers to a family of theories that share historical roots and central tenets, but either diverge in significant ways or otherwise stress different aspects of cognition (Ward et al., 2017). For present purposes, the important aspect of enactivism, as we intend it, is that it emphasizes not only: (1) agent-environment coupling and the importance of bodily morphology for intelligent action, in keeping with other embodied approaches, but also the role of (2) *autonomy* (Varela et al., 1991/2017; Thompson, 2007). Autonomy is here defined as a kind of recursive process of production, in which a system is constituted by a network of processes that recursively depend on each other to generate the processes themselves, and constitute the system as a unity individuated from its environment. To quote Thompson, “an autonomous system is a self-determining system, as distinguished from a system determined from the outside or a heteronomous system” (Thompson, 2007, p. 37). For brevity, we focus on basic metabolic or autopoietic autonomy (Ruiz-Mirazo and Moreno, 2004), i.e., the capacity of a system to reproduce and maintain itself physically. However, enactivists often recognize other forms of autonomy (e.g., neurological, immunological, sensorimotor). Robotics and plant research may benefit from attending to these other forms of autonomy, which find a parallel in the plant kingdom. For instance, in addition to “phytoneural” (Calvo et al., 2017) and sensorimotor behavior, we would do well to examine research in plant immunology (Jones and Dangl, 2006; Li et al., 2020).

Complementing the basic idea of autonomous constitution is the idea that a truly autonomous system is “precarious” — it must actively work to ensure its continued existence. This links autonomy with *adaptivity* (Di Paolo, 2005; see also De Jesus, 2018). Contemporary enactivism places great emphasis on adaptivity—the capacity of the system to actively modify its relationship to the environment in a manner that facilitates its persistence (Di Paolo, 2005; Di Paolo and Thompson, 2014). Marrying autonomy with adaptivity, we get “adaptive autonomy” (Barandiaran, 2002, 2004; Barandiaran and Moreno, 2008; Thompson and Stapleton, 2009), i.e., the notion of a system that regulates its interactions with the world, thereby managing its conditions for viability (the conditions under which it persists as a distinct system). This creates a kind of interdependence between the interaction of a system and its environment and the persistence of that system; actions of a system and its constitution are intertwined.

Although autonomy for enactivists is, strictly speaking, an all or nothing phenomenon—with living systems as the only known instance of an unequivocally autonomous system—we can still think of robots as more or less engineered in relation to enactivist principles. This is because the design of such systems may more or less emphasize autonomy as an important ideal and guiding heuristic (in addition to the importance of morphology and body-environment coupling, shared with other embodied perspectives). Three considerations are worth bearing in mind here. The first is that even embodied robots that are typically thought of as autonomous because they can operate independently for certain durations do not necessarily meet all the requirements for full autonomy in the enactivist sense (Froese and Ziemke, 2009). The second is that even if one falls short of designing a fully autonomous system, autonomy can still function as a model criterion. Finally, a focus on autonomy will produce different results depending on whether research of an individual is animal- or plant-inspired; autonomous growbots may meet different criteria from “locobots” because of their architectural and morphological idiosyncrasies (for a related discussion on the specificity of “organismoid embodiment,” see Vernon, 2010).

Autonomy (as well as adaptivity) is argued to be a crucial determiner of genuine agency. We can unpack agency, from an enactivist perspective, in terms of an autonomous organization that adaptively manages its coupling to the environment and, thus, contributes to sustaining itself (Barandiaran et al., 2009). A more exact definition of “basic autonomy” (which slightly diverges from the traditional formulation in terms of autopoiesis) is provided by Ruiz-Mirazo and Moreno: “the capacity of a system to *manage* the flow of matter and energy through it, so that it can, at the same time, regulate, modify, and control: (i) internal self-constructive processes and (ii) processes of exchange with the environment. Thus, the system must be able to generate and regenerate all the constraints—including part of its boundary conditions—that define it as such, together with its own particular way of interacting with the environment” (Ruiz-Mirazo and Moreno, 2004, p. 240. Original emphasis).

An interesting consequence of the enactivist perspective is that relatively “simple” organisms (including all the higher plants) exhibit genuine agency, whereas robots capable of completing complex information-processing tasks typically do not. Even embodied robots with tight perception-action coupling, though perhaps exhibiting agent-like behavior, do not possess intrinsic agency unless such coupling arises from fulfilling one of that requirements of the system for continued survival (Barandiaran et al., 2009; Stapleton, 2016). In short, enactivism provides relevant perspectives for robotic design concerned with the genuine agency, rooted in the biological processes that are not exclusive to animals. Again, it is important to stress the contrast between the concept of autonomy outlined here and one invoked in many areas of robotics (for discussion on the varied of “autonomy” in robotics, see Smithers, 1997). For example, an “autonomous system” often refers to a robot with the mere capacity to self-manage for some extended period (arbitrarily benchmarked) without human supervision.

Take growth in plant-inspired robotics as a case study (Del Dottore et al., 2018). Enactivism provides the tools to assess the limitations of existing growbots, given its emphasis on homeostatic autonomy (Froese and Ziemke, 2009). Existing robots are capable of growth via root-like appendages, providing novel forms of movement (Sadeghi et al., 2017). Recent examples of effective robotic growth include soft pneumatic robots that achieve directed growth through the pressurization of an inverted thin-walled vessel coupled with controlled asymmetric lengthening, displaying a remarkable ability to move through constrained spaces (Hawkes et al., 2017). However, all the existing forms of plant-inspired roots depend on a prefixed store of energy and matter. Recent pressure-driven robots depend on stored material within a “base station” — a fixed spool of polyethylene tubing provides the material for pressure-driven eversion, i.e., turning inside out—and externally provided source of liquid or air pressure (Hawkes et al., 2017). From an enactivist perspective, a more genuinely autonomous robot actively seeks out and metabolizes all the material for growth in its environment and uses this process to aid its persistence as an individuated system. There are existing robots with artificial digestive systems that seek out energy sources, process them, and egest waste (Melhuish et al., 2006; Ieropoulos et al., 2010). Ecobot-II and -III convert biomass into energy using onboard microbial fuel cells with oxygen cathodes. However, these robots still require an external source to supply key materials.

Moving forward, more truly autonomous growbots—that are plant like in not only their material composition and morphology, but in their adaptive autonomy—will not only self-direct and self-manage in the manner of existing so-called “autonomous” robots (free from direct human management), but will actively seek out the requirements for fulfilling the conditions of their own persistence. This may also be relevant in examining limitations in the *amount* of growth and degree of control possible in existing growbots compared with plants, given their dependence on an external source (Hawkes et al., 2017).

### Value of Plant-Inspired Robots

In addition to any generic benefits afforded by an enactivist perspective—for example, see Smithers (1997) on the role of autonomy for navigating unpredictable environments and Lowe and Kiryazov (2014) on the role of autonomy for cognitive-affective processes—designing robots that are more fully plant like in their material composition, morphology, and autonomous control promises some particular advantages for soft robotics. Obviously, autonomous plant-like robots allow us to test the possibilities of what forms intelligence might assume by taking inspiration from a non-zoological branch on the tree of life. They may also allow us to better test existing theories within plant cognitive science/neurobiology, adopting a “synthetic methodology,” i.e., understanding a phenomenon by building physical systems that simulate aspects of the phenomenon (Pfeifer et al., 2008).

Robots exhibiting more plant-like bodies as well as stronger autonomy also promise practical benefits. These benefits would build upon (but potentially surpass) the advantages of existing plant-inspired robots. This includes the fact that plants display high levels of fault tolerance, with catastrophic damage less likely given the absence of system-critical centralized organs as well as the ability to acquire energy and material in proportion to the demands of growth (a function of their adaptive autonomy). In other words, plants have extensive redundancy built-in to their basic organization. Such a strategy can minimize existential risk (no single root is essential), but it also provides novel ways to reach new locations that have advantages over locomotion (e.g., navigating a hard surface by growing through small cracks). There is also the broad principle that engineering an intelligent system via many “not-so-smart” parts—via principles of swarm intelligence—is often optimal given the cost/risk involved. This is especially relevant, for instance, when designing expensive systems for space exploration (Mehling et al., 2006; Wooten and Walker, 2015; Gallentine et al., 2020).

Designing robots with reference to a more complete suite of plant features including stronger autonomy—thus, has the potential to produce relatively low-cost systems which can be deployed with little configuration and that will actively build themselves while exploring and adapting to their environment with little or no external management. This could have serious implications for space exploration, rescue operations, and medical procedures (see also Blumenschein et al., 2020). Plant-inspired robotics, thus, corroborates the dictum that embodied perspectives both offer theoretical insight into the principles of biological intelligence and are of practical value in the design of adaptive systems (Pfeifer et al., 2008).

To summarize, we suggest there are at least four (overlapping) reasons to consider the design of more holistically plant-inspired robots with strong autonomy as a guiding heuristic:

- To uncover novel forms of robotic design (e.g., “is it possible for a robot to solve problem *x* using a plant-like strategy?”).
- To exploit unique advantages of plant organization for overcoming real-world tasks (e.g., “can plant-like growth afford special benefits for exploring non-terrestrial planets?”).
- To test theories in plant cognitive science/neurobiology (e.g., “can we build a robot with a mechanism analogous to the one we think underlies plant behavior?”).
- To engineer robots that exhibit autonomous, decentralized intelligence as proof of concept for what forms intelligence can take (e.g., “what forms of intelligence are possible to engineer and how similar are these to existing organisms?).

Of course, soft roboticists are already sensitive to some of these considerations, some of the time. As such, recognizing the possibility of more holistically plant-like robots partially serves as a tool to deepen and develop existing trends. Equally, if the preceding discussion is correct, too little attention has been paid to the possibility of genuinely autonomous systems, and the use of strong autonomy as a heuristic to develop more fully plant like (and other autonomous) robots, e.g., robots with more genuinely plant-like growth properties.

Our discussion has explored a two-way relationship between enactivism and the design of more plant-like robots. Enactivism helps us attend to the possibility of looking to plants and other non-zoological sources of inspiration, emphasizing the coupling of adaptive morphology with strong autonomy across the tree of life, while the practical success of plant-inspired robots reinforces a postcognitivist perspective (Heras-Escribano, 2019) on the diverse forms intelligence can take (Linson and Calvo, 2020).

## Conclusion

This article has only begun to unpack the relationship between plant bioinspiration and enactivism. It is apparent, however, that plants offer a rich source of insight for future developments that overlaps with an enactivist perspective and should not be ignored in favor of purely zoological inspiration. Attention to principles of strong autonomy (as exhibited by plants), in conjunction with novel forms of plant-like materials and morphology, might prove beneficial to plant-inspired robotics. It can also serve to assess the limitations of existing plant-inspired robots such as growbots. More broadly, we indicated that an enactive perspective on plant bioinspiration contributes to ensuring that soft robotics is a productive field that generates theoretical insights as well as practical benefits with quantitative advantages. Future research should examine the overlap between the design of more autonomous plant-inspired robots and existing attempts to develop genuinely life-like systems (Kriegman et al., 2020) as well as other postcognitivist perspectives toward plant bioinspiration such as ecological psychology (Frazier et al., 2020). Finally, in addition to issues pertaining to growth and growbots discussed in this article, work on plant-inspired robotics should investigate the potential of development as a key element in more fully plant-like systems, given the significant role of development in plant adaptive behavior (Segundo-Ortin and Calvo, 2021).

## Data Availability Statement

The original contributions presented in the study are included in the article/supplementary material, further inquiries can be directed to the corresponding authors.

## Author Contributions

PC conceived the original idea. PC and JL developed the main concepts. JL wrote the outline and manuscript in consultation with PC. Both authors provided feedback and helped shape the manuscript.

## Funding

This research was supported by the Office of Naval Research Global (Award # N62909-19-1-2015) to PC and the Juan de la Cierva Fellowship from Ministerio de Ciencia e Innovación del Gobierno de España (Award # FJC2019-041071-I) to JL.

## Conflict of Interest

The authors declare that the research was conducted in the absence of any commercial or financial relationships that could be construed as a potential conflict of interest.

## Publisher's Note

All claims expressed in this article are solely those of the authors and do not necessarily represent those of their affiliated organizations, or those of the publisher, the editors and the reviewers. Any product that may be evaluated in this article, or claim that may be made by its manufacturer, is not guaranteed or endorsed by the publisher.
